# The elevated gradient of aversion: a new apparatus to study the rat behavior dimensions of anxiety, fear, and impulsivity

**DOI:** 10.1590/1414-431X20198899

**Published:** 2019-10-28

**Authors:** J.L. Rico, R. Bonuti, S. Morato

**Affiliations:** 1 Laboratory of Animal Behavior, Faculty of Psychology, Fundación Universitaria Konrad Lorenz, Bogotá, Colombia; 2 Laboratório de Comportamento Exploratório, Faculdade de Filosofia, Ciências e Letras de Ribeirão Preto, Universidade de São Paulo, Ribeirão Preto, SP, Brasil

**Keywords:** Anxiety, Exploratory behavior, Impulsivity, Rat, Fear, Self-protection

## Abstract

Few behavioral tests allow measuring several characteristics and most require training, complex analyses, and/or are time-consuming. We present an apparatus based on rat exploratory behavior. Composed of three different environments, it allows the assessment of more than one behavioral characteristic in a short 3-min session. Factorial analyses have defined three behavioral dimensions, which we named Exploration, Impulsivity, and Self-protection. Behaviors composing the Exploration factor were increased by chlordiazepoxide and apomorphine and decreased by pentylenetetrazole. Behaviors composing the Impulsivity factor were increased by chlordiazepoxide, apomorphine, and both acute and chronic imipramine treatments. Behaviors composing the Self-protection factor were decreased by apomorphine. We submitted Wistar rats to the open-field test, the elevated-plus maze, and to the apparatus we are proposing. Measures related to exploratory behavior in all three tests were correlated. Measures composing the factors Impulsivity and Self-protection did not correlate with any measures from the two standard tests. Also, compared with existing impulsivity tests, the one we proposed did not require previous learning, training, or sophisticated analysis. Exploration measures from our test are as easy to obtain as the ones from other standard tests. Thus, we have proposed an apparatus that measured three different behavioral characteristics, was simple and fast, did not require subjects to be submitted to previous learning or training, was sensitive to drug treatments, and did not require sophisticated data analyses.

## Introduction

The elevated plus-maze (EPM) is a model broadly used in the study of anxiety. It was inspired in an experiment reported by Montgomery in the middle of the last century using an elevated Y-maze with a starting enclosed alley leading to a bifurcation (the arms of the Y), where the animals could choose either an arm enclosed by walls or an arm with no walls surrounding it (1). The author reported that, compared with the closed arm, rats explored the open arm less and one of the conclusions was: “Novel stimulation may evoke both the exploratory drive and the fear drive, thus generating approach-avoidance conflict behavior” (ref. 1, p. 260). About thirty years later, the EPM, with two open arms crossed with two enclosed arms, was first reported as a “model of fear-motivated behavior” (2) and validated physiologically and pharmacologically as “a measure of anxiety in the rat” (3): rats with low levels of anxiety would enter and remain longer in the open arms than in the enclosed ones. This explanation reflects a change in standpoint from “approach-avoidance conflict” to “fear-motivated conflict” and/or “anxiety”. Since then, the EPM is considered a good model to study anxiety and it has been investigated not only for entries into the arms but sometimes for many other “ethological” behaviors, such as rearing, stretching, grooming, head-dipping, etc (4).

Later, the focus of studies was on conflict again (5). By comparing rat behavior in three EPMs (a standard one, another with four arms enclosed by walls, and one with all four arms opened), the authors reported that rats, contrary to what was observed in the standard plus-maze, explore all four arms equally when the maze is either totally enclosed or totally open. Thus, they claimed that the typical rat behavior in the standard EPM was determined by conflict when the animals had to leave one arm, passing through the central square, and enter another open or closed arm to explore it. This conclusion led to a research that attempted to reduce conflict by reducing the number of arms in the elevated I-maze, a single elevated alley, half of which was enclosed and the other half open (6). The first trials showed the rats avoided the open arms like in the standard EPM. Initially thought of as an anxiety test, the lack of effect of GABAergic drugs, both an agonist (chlordiazepoxide, five different doses ranging from 0.1 to 5.6 mg/kg) and an antagonist (pentylenetetrazole, four different doses ranging from 1.0 to 30.0 mg/kg), came as a surprise. On the other hand, cyproheptadine (3.0 to 30.0 mg/kg), a serotoninergic, cholinergic, and histaminergic receptor blocker, increased the time spent in the open arm without altering the frequency of entries with the 30.0 mg/kg dose. These drug effects hardly qualify such an apparatus as adequate to study anxiety.

Since the idea of reducing conflict resulted in a model that apparently did not involve anxiety (or at least not only), we reasoned that it would be interesting to attempt to increase conflict: rather than testing open/closed alternatives, we investigated an apparatus with three areas with different motivational properties and elevated from the floor. Thus, the main goal of the present experiment was to investigate the behavior of rats in such an apparatus (see description in the Apparatus section).

## Experiment I – The elevated gradient of aversion: Duration of the session

This experiment was carried out in order to analyze the exploratory behavior of rats in the elevated gradient of aversion (EGA) and to investigate an adequate duration of the testing sessions.

## Material and Methods

### Subjects

Ten male Wistar rats (220±20 g) were obtained from the animal house of the University of São Paulo at Ribeirão Preto, Brazil. They were housed in groups of five in polypropylene cages (41×34×17 cm) with rat chow (Nuvilab, Brazil) and tap water *ad libitum*. The animal room was maintained in a 12-h light/dark cycle (lights on at 7:00 a.m.) with the temperature kept between 24 and 27°C. Cleaning of the cages was performed three times a week and dust-free wood shavings were used as bedding. All testing was performed between 7:30 and 11:30 a.m. All experimental procedures were carried out in accordance with the Guidelines of the Brazilian Society for Neuroscience and Behavior recommendations for animal care and with the U.K. Animals (Scientific Procedures) Act 1986, and associated guidelines.

### Apparatus

**Figure 1.**
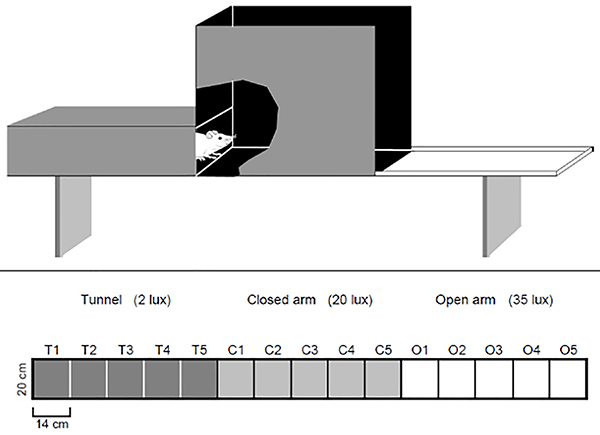
Schematic representation (upper part) and a top view of the EGA (lower part). Dark gray indicates the tunnel compartment with area division (T1 to T5); light gray indicates the closed arm compartment (C1 to C5); and white indicates the open arm compartment (O1 to O5).

The apparatus was composed of three compartments equal in size with different motivational properties. It consisted of a 210×20-cm alley divided into three 70×20-cm distinct areas (Figure 1, top). The first compartment at one extremity of the apparatus (used as the starting point) was a tunnel, closed by 25-cm high walls and covered with transparent red Plexiglas (which allowed observation and video recording), with floor and walls lined with black opaque Formica laminate. At the extremity of the apparatus, there was a sliding door (20×25 cm) through which the subjects were introduced into the apparatus tunnel and which was the starting point. The other end of the tunnel communicated with the second (middle) compartment, which was surrounded with 60-cm high walls. Both the floor and the walls were lined with black opaque laminate, resembling a closed arm of an EPM. At the opposite end, the tunnel communicated with the third compartment, which had no walls, was lined with white opaque laminate, and was surrounded with a 1-cm high white rib to prevent rats from falling off the apparatus, resembling an open arm of an EPM. The whole set was elevated 50 cm from the floor.

### Procedures

Each subject was individually placed in the extremity of the tunnel opposed to the passage to the closed arm, then the sliding door was closed and the animal was allowed to freely explore for 120 min. After each session, the apparatus was cleaned with a 5% ethanol solution and dried with a cloth. The tests were carried out in a room lit by a 60-W incandescent bulb 2.7 m above the floor. It provided illumination to the test room and allowed the following light intensities measured at the center of each compartment: 2 lux inside the tunnel, 20 lux inside the closed arm and 35 lux in the open arm.

All behavioral tests were recorded by a video camera placed above the EGA and connected to a video recorder in an adjacent room. Videos were subsequently analyzed by a trained observer. Similar to a previously described procedure (7), behavioral parameters were scored with a behavior scoring freeware (X-PloRat) developed at the Laboratory of Exploratory Behavior, University of São Paulo at Ribeirão Preto, Brazil (8). To record the behavior of the subjects, the EGA floor image was divided into fifteen 14×20-cm rectangles (Figure 1, lower part) on a transparent plastic mask placed over the computer screen. The number of entries and time spent in the different compartments and in the rectangles of the EGA were scored. The latency to enter the open arm for the first time was also scored. An entry into a compartment or into a rectangle within a compartment was scored after all four paws of the rat entered it.

### Data analysis

The data obtained are reported as median and interquartile range and the number of transitions was analyzed by nonparametric Friedman repeated measures test followed by the Dunn’s test, when appropriate. In all cases, the level of significance was set at P<0.05.

## Results

Preliminary observation of the videos revealed that all exploratory activity occurred in the first 60 min; after that, the rats remained immobile inside the tunnel. Thus, we analyzed only the first half of the session, divided into three parts: 0 to 3, 4 to 15, and 16 to 60 min. Statistical analysis showed significant differences between the intervals for entries both into the closed arm (χ^2^=17.20, P<0.001) and into the open arm (χ^2^=8.00, P<0.05). *Post hoc* comparisons indicated the animals entered both the closed and open arms more times during the first 3 min, compared to the other two intervals. It is interesting to note that no entries into the open arm were observed from the 4th min to the end of the session (Table 1).

**Table 1:** Frequency of closed and open arm entries by rats during the first 60 min of the session.

Session intervals (min)	Median	Interquartile ranges
Closed arms		
1–3^*^	3	(3–3)
4–15	1	(1–2)
16–60	1	(1–2)
Open arms		
1–3^*^	0	(0–1)
4–15	0	(0–0)
16–60	0	(0–0)

*P<0.05 compared to 4–15 and 16–60 (Friedman and Dunn’s *post hoc* test).

## Discussion

The data demonstrated clearly that exploratory behavior of the whole apparatus tended to occur in the first 3 min of the session. Thus, we chose this duration for all sessions involving this apparatus from then on.

## Experiment II – The elevated gradient aversion: Factor analysis

After determining that a 3-min session was enough for rats to explore both the closed and open arms, the present experiment was carried out to investigate the different behavioral dimensions elicited by exposing rats to the EGA.

## Material and Methods

### Subjects and apparatus

Sixty male Wistar rats with the same characteristics described in Experiment I, as well as the same apparatus and test room, were used.

### Procedures

Each subject was gently placed into the EGA through the sliding door in the tunnel extremity and allowed to explore the apparatus for 3 min. The following parameters were analyzed: time spent in the compartments, time spent in each rectangle, latency of the first entry into the open arm, and distance traveled (estimated by multiplying the number of rectangles crossed by the width of the rectangles: 0.14 m).

### Data analysis

The data from all animals were subjected to two successive factor analyses. In order to identify the main spatiotemporal measure of the EGA, the first factor analysis was performed on the time spent in each of the 15 rectangles. In the second factor analysis, the outcome from the first analysis was combined with the following measures: closed and open arm entries, latency of the first entry into the open arm, and distance traveled. The factor analyses were performed by principal component analysis followed by an orthogonal Varimax rotation. Factors with eigenvalues greater than 1 and loadings greater than 0.5 were kept.

## Results

**Figure 2.**
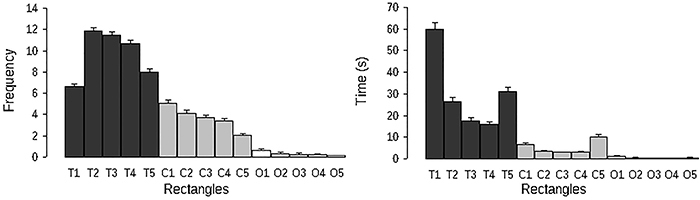
Frequency of entries (left) and time spent (right) in the rectangles of the elevated gradient of aversion apparatus. T1-T5, Tunnel section; C1-C5, closed arm section; O1-O5, open arm section.

Figure 2 shows the frequency of entries into and the time spent in each rectangle of the EGA. Results from the first factor analysis are shown in Table 2. Measure of time spent in each of the 15 rectangles were subjected to principal component analysis. After an orthogonal Varimax rotation, five factors emerged representing 68.45% of the variance. The first two factors that accounted for 40.04% of the variance were related to the time spent in the closed and open arms. The time spent in the five rectangles of the tunnel contributed to the emergence of the last three factors. The time spent in the five rectangles of the closed arm correlated positively with factor 1, while the time spent in the rectangles of the open arm loaded on factor 2. Time spent in the first (T1) and second (T2) rectangles of the tunnel correlated negatively within factors 3 and 4, respectively. Factor 5 was only related to the time spent in the last rectangle of the tunnel (T5), closer to the closed arm. The closed end of the tunnel (T1) is the only rectangle of the alley protected by five surfaces (three walls, roof, and floor) and the time spent in T1 and T2 loaded modestly (eigenvalue <0.5) with time spent in areas outside the tunnel, which suggested that T1 and T2 represented a protected area for the rats. Factor analysis 1 revealed four main areas of the EGA: a safe area (T1 and T2), a transition area (T5), which allowed entry into the closed arm, and the closed and the open arms.

**Table 2:** Orthogonal factor loadings for time in the rectangles, frequency of entries, and time spent in the 15 rectangles into which the elevated gradient of aversion test floor was divided.

Rectangle	Factors				
	F1	F2	F3	F4	F5
Tunnel					
T1			–0.683		
T2				–0.821	
T3			0.789		
T4			0.736		
T5					-0.931
Closed arm					
C1	0.555				
C2	0.741				
C3	0.724				
C4	0.788				
C5	0.783				
Open arm					
O1					
O2		0.737			
O3		0.764			
O4		0.799			
O5		0.825			
% variance	25.47	14.58	11.12	10.07	7.22

Principal component analysis followed by an orthogonal Varimax rotation. Factors with eigenvalues greater than 1 and loadings greater than 0.5 were kept. Minus signs indicate the direction of the loading.

The second factor analysis was performed to identify the relationship between the four spatiotemporal measures obtained from the first factor analysis and the following measures: frequency of entries into the closed and open arms, latency of the first entry into the open arm, and total distance traveled in the apparatus. Results from the second factor analysis are reported in Table 3. Three factors emerged after an orthogonal Varimax rotation, which accounted for 79.31% of the total variance. Closed arm exploration measures and total distance traveled loaded on factor 1. Time spent in the safe area of the tunnel also loaded on this factor, but negatively. Factor 2 was associated with the exploration of the more aversive area: the open arm. Frequency of entries and time spent in the open arm loaded positively on factor 2, whereas latency of the first entry into the open arm loaded negatively on the same factor. Finally, factor 3 was related with time spent in the tunnel areas. Time in the transition (T5) area loaded negatively, whereas time in the safe area (T1 and T2) loaded positively.

**Table 3:** Orthogonal factor loadings for main spatiotemporal and behavioral measures of the elevated gradient of aversion test.

Behavioral parameters	Factors		
	F1	F2	F3
Time in safe area (s)	–0.619		0.659
Time in transition area (s)			–0.898
Time in closed arm (s)	0.904		
Closed arm entries (n)	0.893		
Time in open arm (s)		0.885	
Open arm entries (n)		0.945	
Latency to entry open arm (s)		–0.938	
Distance traveled (m)	0.851		
% variance	45.20	19.94	14.16

Principal component analysis followed by an orthogonal Varimax rotation. Factors with eigenvalues greater than 1 and loadings greater than 0.5 were kept. Minus signs indicate the direction of the loading.

A description of the behavioral profile of rats exposed to the EGA for 3 min is shown in Table 4. The animals remained 47.8 and 17.25% of the session in the safe and in the transition areas of the tunnel, respectively. All rats entered into the closed arm and spent 14.84% of session in this compartment. Only 20 of 60 rats entered the open arm and spent 1.52% of session in that compartment.

**Table 4:** Description of the behavioral profile of the rats exposed to elevated gradient of aversion test for 3 min.

Behavioral parameters	Mean±SE
Time in safe area (s)	86.04±3.36
Time in transition area (s)	31.06±1.91
Time in closed arm (s)	26.72±1.87
Closed arm entries (n)	2.82±0.16
Time in open arm (s)	2.73±0.59
Open arm entries (n)	0.43±0.09
Latency to entry the open arm (s)	131.96±9.35
Distance traveled (m)	9.60±0.34

Behavioral parameters were obtained from factor analyses reported in Tables 1 and 2.

## Discussion

Factor 1 described the rats' tendency to leave the safe area (time loaded negatively) and to explore the rest of the tunnel and the closed arm (frequency of entries and time spent in the closed arm and total distance traveled), suggesting this factor may be related to exploratory behavior. Factor 2 indicated the rats explored the open arm (frequency of entries and time spent) and entered it more readily (latency to enter loading negatively), suggesting it may be related to behavioral disinhibition, or impulsivity, since the animals promptly entered the less safe part of the apparatus but promptly left it, remaining very little time in the open arm. Factor 3 indicated the animals spent more time in the safest area of the apparatus (tunnel extremity) and less in the transition area (T5), suggesting it may be related to self-protection, since the animals took longer to leave this area. These labels, however, are used more in a descriptive than in an explanatory way.

It is difficult to ascertain the motivational states driving the behaviors that compose each of these three factors. In factor 1 (Exploration), the rats could be just exhibiting exacerbated motor activity. However, on the other hand, they could be exploring for information acquisition about the new environment. Besides that, the behavior could also be due to an anxiety level low enough as to allow the animals to explore and move about. In other tests measuring exploratory behavior, decreases in the levels of anxiety increase exploration. For example, anxiolytic drugs cause rats to increase exploratory behavior in the open field (OF) test (9) and in the EPM (10). However, both motivational states, information acquisition and decreased level of anxiety, could be acting in combination, resulting in the behaviors that loaded in this factor.

Factor 2 (Impulsivity) included the behavior of animals that ventured to visit the open arm and did so very rapidly. Also, they visited the open arm repeatedly despite having already acquired information about the aversive characteristics of that compartment. This hinted at both a rapid decision-making process to move from the tunnel to the open arm, and an increased exposure to risk. This behavioral repertoire resembled some of the impulsivity definitions including: 1) inefficiency to withhold or stop a response in spite of adverse consequences, 2) novelty-seeking, and 3) an increased propensity to engage in risky behaviors (11). Besides that, again, such behavior is probably due to a low anxiety level that allow the animals not to freeze and rapidly enter the open arm. But, again, it could also be due to elevated motor activity. In studies with the step-down test, however, shorter latencies are indicative of lower levels of anxiety (12). In addition, some authors consider shorter latencies to enter the center of an OF as a good measure of impulsivity (13). Nevertheless, ascertaining impulsivity in a short 3-min session is advantageous, since behavioral operant tasks proposed for that objective involve previous learning and/or are usually time-consuming (11). The exploratory behavior-based test we propose does not demand previous training and animals rely on voluntary risk-taking behaviors rather than learning tasks. Impulsive-related measures obtained from a free-exploration procedure have the advantage of avoiding the possible interpretation of learning deficit as impulsivity (14).

Likewise, in factor 3 (Self-protection), the resulting behavior could be motivated by anxiety or fear in general or just avoidance of open spaces. It should be noted that the two measures composing this factor occurred in the tunnel area, the most protected place in the EGA. A decrease in the anxiety level as well as a decrease in fear would conceivably allow the animals to leave this area.

Taken together, the results from the second factor analysis suggested the EGA could identify a tendency to explore a novel environment (factor 1) and both a disinhibition, or impulsive characteristic (factor 2), as well as a self-protection-related characteristic (factor 3) of naive rats in a short 3-min session. The results also did not discard the possibility that anxiety levels are involved in behaviors that compose each of the three factors. However, an animal exhibiting exacerbated motor activity could also provide wrong insights regarding factors 1 to 3. One way to circumvent this problem is to correlate EGA measures with measures of motor behavior obtained with other tests used to assess these behaviors. Another way to investigate these hypotheses included in the proposal of factors 1 to 3 is to test the animals under the effect of behavior-affecting drugs. Experiment III compared the behavior of rats submitted to the EGA, EPM, and OF tests. Finally, Experiment IV investigated behavioral drug effects using the EGA test.

## Experiment III – Behavioral comparison of rats submitted to the OF, EPM, and the EGA

Several reports in the literature state that both the OF and the EPM tests yield measures related to motor activity/exploration and anxiety/fear/emotionality. In the OF, fear (or emotionality or anxiety) is usually considered to be correlated with decreased time spent in the center of the apparatus while motor activity (or exploration) is considered to be related to ambulation (for a review, see Walsh and Cummings (9). However, some researchers consider ambulation to also be related to anxiety (15). In the EPM, anxiety is usually considered to be correlated with decreased time spent in the open arms of the apparatus while exploration is considered to be related to ambulation, most frequently in the closed arms (for a review, see Carobrez and Bertoglio (10).

Both the OF and the EPM have been subjected to factor analysis studies. In spite of some differences in the number of factors, there are common points in these studies. Most factor analysis studies using rats and the OF test present at least two factors, which include behavioral measures considered either related to exploratory/motor activity or emotionality/fear/anxiety (16–18). Factor analysis studies using the EPM and rats also present at least two factors, which include behavioral measures interpreted either as anxiety or exploration (4,19).

The goal of the present experiment was to measure behaviors in the OF, EPM, and EGA and correlate EGA factor measures with measures involving exploratory/motor activity and emotionality/fear/anxiety in the OF and anxiety and exploration in the EPM. Mainly, we were interested in determining correlations between ambulation in the OF and in the EPM with the latency to enter the open arm in the EGA (Impulsivity factor), since positive correlations would indicate entries in this arm are linked to ambulation and a lack of correlation would favor our hypothesis of entries into the open arm as being due to impulsivity. Also, we were interested in determining correlations between anxiety/fear/emotionality in the OF and EPM with the measures that constitute the self-protection factor in the EGA, since positive correlations would indicate self-protection measures are linked to anxiety and a lack of correlation would favor our hypothesis that this factor is something other than anxiety, perhaps fear.

## Material and Methods

### Subjects and apparatus

Thirty male Wistar rats with the same characteristics as described in Experiment I were used.

We used a wooden OF (120×120×40 cm) lined with dark brown opaque laminate. The EPM consisted of two open arms (50×10 cm) crossed at right angles with two opposed arms of the same size enclosed by dark brown wooden walls (40 cm high), except for the central part where the arms crossed. The apparatus was elevated 50 cm above the floor. To prevent the rats from falling, a rim of Plexiglas (0.5 cm high) surrounded the perimeter of the open arms. The EGA apparatus was the same as described in Experiment I.

### Procedures

All tests were conducted in the same test room as the previous experiments, between 08:00 and 11:30 a.m. On the first two days, the rats were tested in the OF in 5-min sessions. There was no session on the third day. On the fourth and fifth days, they were tested in the EPM in 5-min sessions. There was no session on the sixth day. On the seventh day, they were tested in the EGA in 3-min sessions.

All behavioral tests were recorded by a video camera placed above the apparatuses and connected to a video recorder in an adjacent room. Videos were subsequently analyzed by a trained observer. Behaviors were scored with the X-PloRat software. To record where the behaviors occurred, the OF floor image was divided into 36 20-cm squares on a transparent plastic mask placed over the computer screen, while the EPM floor image was divided into 21 10-cm squares. Recording of EGA sessions were done as described in previous sessions.

For the OF test, rats were gently place in the center of the apparatus and allowed to freely explore for 5 min. The behaviors recorded were: time spent in the center and in the corners and the number of crossed squares, which allowed estimating the distance traveled in meters. For the EPM, rats were gently placed in the central square and allowed to freely explore for 5 min. The behaviors recorded in the EPM were time spent in the open arms and the total square entries, which allowed estimating the distance traveled in meters. For the EGA, rats were gently placed into the sliding door of the tunnel and allowed to freely explore for 3 min. The behaviors recorded in the EGA were those that compose factors 1 to 3.

### Data analysis

Pearson correlation test was used to analyze the measures obtained with the three apparatuses. In all cases, a significance level of P<0.05 was used.

## Results

Table 5 presents the behavioral measures (means±SE) in the three apparatuses and the correlation coefficients with the probabilities shown between parentheses. Two EGA measures (closed arm entries and total distance traveled) correlated positively with the total distance traveled in the OF. In addition, time spent in the corners of the OF correlated negatively with both the time spent in the center and the distance traveled in this apparatus. Finally, total distance traveled in the EPM correlated negatively with time spent in the center of the OF.

**Table 5:** Correlation indices between open-field (OF) and elevated plus-maze (EPM) measures of ambulation and anxiety with elevated gradient of aversion (EGA) measures.

Measures	Mean±SE	OF			EPM	
		Corners (s)	Center (s)	Distance (m)	Open arm (s)	Distance (m)
EGA						
1,3 - Safe area/Time (s)	61.8±4.2	0.196 (0.298)	0.196 (0.298)	0.238 (0.206)	0.124 (0.512)	0.181 (0.338)
1 - Closed arm - Time (s)	55.0±4.7	–0.162 (0.362)	0.072 (0.703)	0.176 (0.353)	0.019 (0.920)	–0.081 (0.669)
1 - Closed arm entries	24.0±2.2	–0.255 (0.174)	0.011 (0.954)	**0.460 (0.011)**	–0.036 (0.852)	–0.119 (0.531)
1 - Distance traveled (m)	9.2±0.6	–0.313 (0.092)	0.087 (0.646)	**0.524 (0.003)**	–0.111 (0.560)	0.042 (0.825)
2 - Latency/Time (s)	137.5±11.2	0.210 (0.264)	–0.162 (0.392)	–0.069 (0.718)	–0.177 (0.351)	0.186 (0.326)
2 - Open arms/Time (s)	4.4±1.5	–0.033 (0.863)	0.157 (0.408)	–0.074 (0.699)	0.149 (0.432)	–0.140 (0.459)
2 - Open arm entries	1.1±0.3	0.166 (0.369)	0.180 (0.342)	0.112 (0.554)	0.149 (0.431)	–0.128 (0.501)
3 - Transition area/Time (s)	37.8±4.1	0.166 (0.369)	–0.169 (0.371)	0.155 (0.414)	0.119 (0.530)	0.042 (0.825)
OF						
Corners/Time (s)	184.4±8.0	-	-	-	-	-
Center/Time (s)	28.7±4.4	**–0.663 (<0.001)**	-		-	-
Distance traveled (m)	16.0±1.7	**–0.568 (<0.001)**	0.083 (0.664)	-	-	-
EPM						
Open arms - Time (s)	50.0±9.3	0.019 (0.920)	0.069 (0.718)	–0.214 (0.255)	-	-
Distance traveled (m)	6.0±0.6	0.344 (0.062)	**–0.452 (0.012)**	0.273 (0.145)	–0.044 (0.816)	-

Data are reported as time in seconds or distance traveled in meters. Probability values are shown between parentheses below the correlation coefficients (Pearson’s product moment correlation test). P<0.05 values are shown in bold.

## Discussion

Experiment II had proposed three factors to explain rat behavior in the EGA. These factors included behaviors related to exploration, impulsivity, and self-protection.

The significant correlation between factor 1 of EGA measures (closed arm entries and distance traveled) and OF motor activity factor (distance traveled) indicated the rats' displacements in both apparatuses may be related to information acquisition, suggesting exploratory behavior. These EGA measures, however, did not present positive correlations with displacements in the EPM. The fact that these displacements also did not correlate with OF displacements suggested that EPM displacements are not only motivated by information-seeking but by other types of motivation. Thus, displacements in the OF and EGA were indicative of exploration to obtain information about the novel environment.

The measures included in EGA factor 2 (Impulsivity) did not correlate with any measure of either the OF or the EPM. This indicated that the factor was not related to either anxiety or to exploratory behavior. In addition, considering its characteristics of rapid and frequent entering into the open arm and the little time spent in it, it was plausible to consider factor 2 as indicative of impulsivity.

The measures included in EGA factor 3 (Self-protection) also did not correlate with any measure of either the OF or the EPM. This indicated that the factor was also not related to either anxiety or to exploratory behavior. Also, considering the characteristic of remaining inside the tunnel, it was plausible to consider that factor 3 was more fear-motivated than anxiety-motivated.

It is difficult to be sure about the motivations of rats exploring the three different novel environments but the results allowed us to discard elevated motor activity as having an influence on EGA factors 2 and 3. Also, the results did not allow us to discard anxiety as having an influence on EGA factors 1 to 3. Anxiety may be present in these situations interacting with other factors. Increased distance traveled and closed arm exploration (EGA factor 1) in the EGA may be due to anxiety the same way as increased ambulation in new environments, as suggested by Welker (15). Prompt and frequent entries into the open arm (EGA factor 2) may occur when the animals are not able to restrain from entering potentially dangerous places, as discussed by Beuzen and Belzung (12), Pawlack et al. (13), and Bari and Robbins (11). Permanence in protected areas of the tunnel (EGA factor 3) may be fear-motivated rather than connected to anxiety. Thus, the EGA may be an alternative to measure and investigate exploration, impulsivity, and self-protection.

In order to better understand the behaviors included in the factors, the next experiment investigated drug effects on rats tested in the EGA.

## Experiment IV – Effects of chlordiazepoxide, pentylenetetrazole, apomorphine, and imipramine on the behavior of rats submitted to the EGA

Once three behavioral dimensions for the EGA were established, the present experiment was carried out to investigate the effect of pharmacological treatments on the behaviors correlated with the dimensions of the test. Since the GABAergic, dopaminergic, and serotonergic systems are implicated in the expression of impulsive behavior (20–22), this experiment investigated the effects of acute treatment with the benzodiazepine chlordiazepoxide (CDP), the anxiogenic drug pentylenetetrazole (PTZ), and the non-selective dopaminergic agonist apomorphine hydrocliride (APO). The effects of acute and chronic treatment with the tricyclic antidepressant imipramine (aIM and cIM) were also analyzed.

## Material and Methods

### Subjects and apparatus

One hundred and thirty male Wistar rats with the same characteristics described in Experiment I were used. The EGA apparatus was the same described in Experiment I.

### Procedures

All subjects were submitted to 3-min EGA sessions. Test conditions, subject handling, and data recording were the same as in the previous experiments. On the test day, groups of rats (n=10) were randomly allocated to the drug treatments and received intraperitoneal (*ip*) injections before the sessions.

### Pharmacological treatments

In all administrations, a volume of 1 mL/kg of drug solution or control vehicle was injected. CDP (0, 5, and 10 mg/kg, Deg, Brazil) was suspended in distilled water containing a drop of Tween 20 and injected 30 min before the test. Distilled water with a drop of Tween 20 was used as control vehicle. PTZ (0, 10, and 20 mg/kg, Sigma-Aldrich, USA) was dissolved in 0.9% NaCl solution (also used as control vehicle) and injected 5 min before the test. APO (0 and 1 mg/kg, Sigma-Aldrich) was dissolved in 0.9% NaCl solution (used as control vehicle) and injected 15 min before the test. IM (Liane Manipulação, Brazil) was dissolved in 0.9% NaCl solution (used as control vehicle) and injected either 30 min before the test (aIM) or chronically (cIM) in 20 daily *ip* injections followed by an *ip* injection 30 min before the test. Acute treatment included three doses (0, 8, and 16 mg/kg) while chronic treatment included two doses (0 and 16 mg/kg). Drugs, doses, and timing were chosen based on previous rat studies (23,24).

### Data analysis

Data from the pharmacological treatments are reported as means±SE and analyzed by one-factor analysis of variance (ANOVA) and followed, when appropriate, by Duncan's *post hoc* pairwise multiple comparison test. When data were non-normally distributed or when variance was heterogeneous, the nonparametric Kruskal-Wallis test was used and followed, when appropriate, by Dunn's pairwise multiple comparison test. Whenever one dose was studied, control and treatment groups were compared by Student's *t*-test or the Mann-Whitney rank sum test depending on normality and variance homogeneity.

## Results

**Figure 3.**
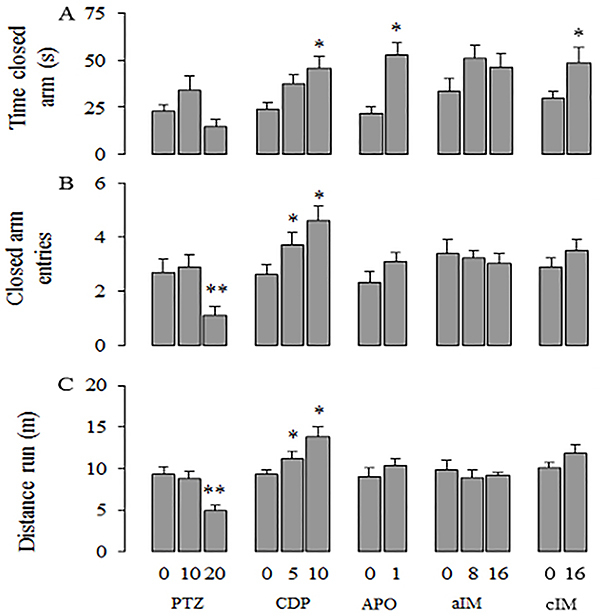
Effects of pentylenetetrazole (PTZ), chlordiazepoxide (CDP), apomorphine (APO), acute imipramine (aIM), and chronic imipramine (cIM) on the first factor (Exploration). Data are reported as means±SE. *P<0.05 compared to control vehicle (P<0.05); **P<0.05 compared to the other dose. The following statistical tests were used: one-way ANOVA followed by Duncan’s *post hoc* test, Kruskal-Wallis followed by Dunn’s *post hoc* test or Student’s *t*-test (for details, see Results section of Experiment IV).

**Figure 4.**
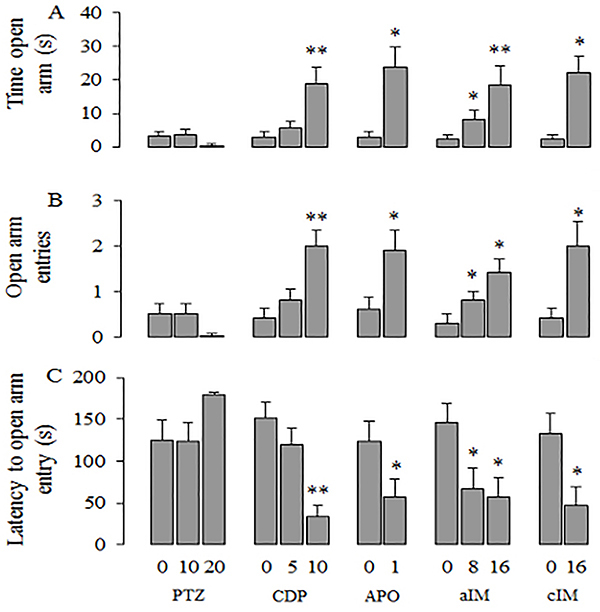
Effects of pentylenetetrazole (PTZ), chlordiazepoxide (CPD), apomorphine (APO), acute imipramine (aIM), and chronic imipramine (cIM) on the second factor (Impulsivity). Data are reported as means±SE. *P<0.05 compared to control vehicle; **P<0.05 compared to the other doses. The following statistical tests were used: one-way ANOVA followed by Duncan’s *post hoc* test, Kruskal-Wallis followed by Dunn’s *post hoc* test, Student’s *t*-test, or Mann-Whitney test (for details, see Results section of Experiment IV).

**Figure 5.**
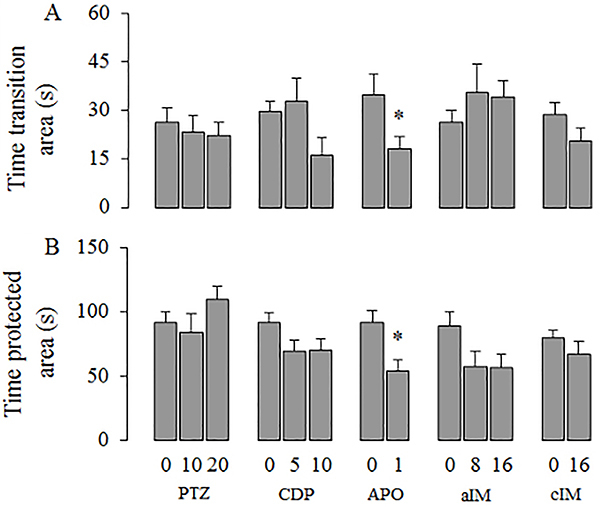
Effects of pentylenetetrazole (PTZ), chlordiazepoxide (CPD), apomorphine (APO), acute imipramine (aIM), and chronic imipramine (cIM) on the third factor (Self-protection). Data are reported as means±SE. *P<0.05 compared control vehicle. Statistical analysis was done with Student’s *t*-test.

Figure 3 presents the effects of chlordiazepoxide, pentylenetetrazole, apomorphine, and acute and chronic imipramine on the measures loading in factor 1 (Exploration). Figures 4 and 5 present the effects of these drugs on the measures loading on factor 2 (Impulsivity) and factor 3 (Self-protection), respectively.

### Effects of CDP and PTZ

Behavioral parameters with positive loadings on factors 1 and 2 (see Table 3) were significantly increased by CDP: time in the closed arm (F [2,29]=4.420, P=0.022), closed arm entries (F [2,29]=6.972, P=0.004), distance traveled (H (2]=7.301, P=0.026] (Figure 3), time in open arm (H [2]=12.099, P=0.002), and open arm entries (H [2]=12.037, P=0.002; Figure 4). *Post hoc* tests showed that 10 mg/kg of CDP increased all measures loading in factors 1 (Exploration) and 2 (Impulsivity). The dose of 5 mg/kg of CDP increased the measures of closed arm entries and distance traveled. Latency to enter the open arm, which loaded negatively on factor 2, was significantly decreased by CDP (H [2]=16.031, P=0.001; Figure 4C). Latency was decreased by 10 mg/kg but not by 5 mg/kg. Measures with loadings on factor 3 (Self-protection) were not affected by CDP (Figure 5).)

Only two measures with positive loadings on factor 1 (see Table 3) were significantly decreased by PTZ: closed arm entries (F [2,29]=5.562, P=0.009;) and distance traveled (F [2,29]=7.293, P=0.003; Figure 3). *Post hoc* tests showed that 20 but not 10 mg/kg of PTZ reduced these two measures. Measures loading on factors 2 and 3 were not affected by PTZ (Figures 4 and 5).

### Apomorphine effects

Behavioral measures with positive loadings on factor 2 (see Table 3) were significantly increased by APO as shown by the time spent in the open arm (U=85.00, P=0.008) and by open arm entries (U=78.00, P=0.030), while latency to enter the open arm, which loaded negatively on the same factor, was not significantly altered by APO (U=30.00, P=0.128; Figure 4). Measures correlated with factor 3 were only affected by APO: rats significantly reduced both the time spent in the transition area (t [18]=2.231, P=0.039) and in the safe area (t [18]=3.144, P=0.006; Figure 5). Although time spent in the closed arm (t [18]=–4.106, P<0.001) and closed arm entries (t [18]=–2.893, P=0.010] were significantly increased by APO, distance traveled in the close arm (t [18]=–1.001, P=0.330; Figure 3) was not altered.

### Imipramine effects

Measures with positive loadings on factor 2 (see Table 3) were significantly increased by both the acute (time spent in the open arm: H [2]=9.480, P=0.009, Figure 4A and open arm entries: H [2]=7.239, P=0.027, Figure 4B) and chronic IM treatments (time spent in the open arm: U=85.00, P=0.008, Figure 4A and open arm entries: U=81.50, P=0.038, Figure 4B). In contrast, the latency to entry into the open arm, with negative loading on the same factor, was significantly decreased by both acute (F [2,29]=4.473, P=0.021; Figure 4C) and chronic treatments (t [18]=2.605, P=0.018, Figure 4C). The remaining parameters that loaded on factors 1 and 3 were not affected by the acute or by the chronic treatments with IM.

## Discussion

### Factor 1 – Exploration

Several drugs have long been known to facilitate laboratory animal behavior by increasing its frequency of responses in operant or naturalistic tasks (for a review, see Cook et al. (25)). Psychostimulants like amphetamine or cocaine (which enhance dopamine effects in the synaptic cleft) increase rat bar pressing and motor activity in a variety of experimental situations (26). An analogous effect is caused by minor tranquilizers (for a review, see Sanger et al. (27)), which also increase rat bar pressing and ambulation in both the OF and the EPM (10,28). Similar increases were obtained in the present experiment: most measures loading on factor 1 were enhanced by APO and CDP. Conversely, PTZ decreased closed arm entries and distance traveled but did not significantly affect time spent in the open arm. Imipramine, on the other hand, did not alter measures loading on factor 1. These results agree with other negative ones obtained in rats exposed to the plus-maze and treated with IM either acutely (3,29) or chronically (30). Taken together, the effects of CDP, PTZ, and APO seem to indicate that only motor activity enhancement is not enough to explain how the measures loading on factor 1 were altered. For example, APO, a drug that enhances motor activity, did not alter distance traveled. PTZ altered all these measures with the exception of time spent in the closed arm. This suggests this factor may be related to something more complex than just motor activity. Exploratory behavior may be a good explanation for this factor, and as such, the motivation for this set of behaviors may be complex, involving curiosity or information seeking (increased ambulation), fear (PTZ effect), and simple motor activity.

### Factor 2 – Impulsivity

The data obtained in the present experiment agreed with those observed in rats exposed to the EPM. It has been shown that APO increases the time spent in the open arms of the maze via the dopamine D2 receptors (23,31). This also agrees with the claim that open-arm exploration in the EPM can be used as an index of impulsive behavior (32). In addition, it has been reported that apomorphine treatment leads patients with parkinsonism to develop impulse-control disorders (33,34). Also, Wistar rats treated with apomorphine exhibit both attentional deficits in operant tasks (35) and disrupted information processing in the prepulse inhibition paradigm (36,37). Our results suggested that a high dose of CDP increasesd impulsivity-related behaviors in the EGA, which agreed with the effect of chlordiazepoxide on impulsive measures obtained in rats exposed to operant tasks (20–22). This suggested this factor to be related to impulsivity and/or lack of control.

### Factor 3 – Self-protection

In discussing the data from Experiment II, we hypothesized that the two measures composing factor 3, involving remaining in the tunnel area, could be motivated by either anxiety or fear. However, in Experiment III, these two measures did not correlate with measures from the OF and EPM, usually considered indices of anxiety. Thus, it is conceivable that the two behavioral measures that compose factor 3 are fear-motivated rather than anxiety-motivated. Apomorphine was the only compound that changed the measures loaded on this factor. Rats treated with this non-selective dopamine agonist spent less time both in the safe area of the tunnel and in the transition area between the tunnel exit and the closed arm. Traditionally, some dopaminergic mechanisms have been associated to increased motivation to explore and approach environmental stimuli (38–40). Thus, it may be possible that this drug increased the motivation to explore and inhibited fear, leading to the decrease in the time spent in these areas of the tunnel.

### Conclusion

Briefly, the results suggested that the EGA can be a useful model for screening exploratory, impulsive, and fear traits in rats exposed to this novel environment. The 3-min session duration was clearly an advantage. The above interpretations were reinforced by the correlations with measures from other apparatuses and by our pharmacological study. Further studies are necessary in order to better analyze the behavioral response of animals and confirm that the three factors presented here are indicative of the motivations that were proposed.
